# GapBlaster—A Graphical Gap Filler for Prokaryote Genomes

**DOI:** 10.1371/journal.pone.0155327

**Published:** 2016-05-12

**Authors:** Pablo H. C. G. de Sá, Fábio Miranda, Adonney Veras, Diego Magalhães de Melo, Siomar Soares, Kenny Pinheiro, Luis Guimarães, Vasco Azevedo, Artur Silva, Rommel T. J. Ramos

**Affiliations:** 1 Institute of Biological Sciences, Federal University of Pará, Belém, Pará, Brazil; 2 Institute of Biological and Natural Sciences, Federal University of Triângulo Mineiro, Uberaba, Minas Gerais, Brazil; 3 Institute of Biological Sciences, Federal University of Minas Gerais, Belo Horizonte, Minas Gerais, Brazil; Georgia Institute of Technology, UNITED STATES

## Abstract

The advent of NGS (Next Generation Sequencing) technologies has resulted in an exponential increase in the number of complete genomes available in biological databases. This advance has allowed the development of several computational tools enabling analyses of large amounts of data in each of the various steps, from processing and quality filtering to gap filling and manual curation. The tools developed for gap closure are very useful as they result in more complete genomes, which will influence downstream analyses of genomic plasticity and comparative genomics. However, the gap filling step remains a challenge for genome assembly, often requiring manual intervention. Here, we present GapBlaster, a graphical application to evaluate and close gaps. GapBlaster was developed via Java programming language. The software uses contigs obtained in the assembly of the genome to perform an alignment against a draft of the genome/scaffold, using BLAST or Mummer to close gaps. Then, all identified alignments of contigs that extend through the gaps in the draft sequence are presented to the user for further evaluation via the GapBlaster graphical interface. GapBlaster presents significant results compared to other similar software and has the advantage of offering a graphical interface for manual curation of the gaps. GapBlaster program, the user guide and the test datasets are freely available at https://sourceforge.net/projects/gapblaster2015/. It requires Sun JDK 8 and Blast or Mummer.

## Introduction

Next generation sequencing (NGS) platforms have reduced sequencing costs and increased the amount of data generated, resulting in a greater number of complete genomes for eukaryotes and prokaryotes, which are subsequently deposited in public databases [1,2].

Several computational tools have been developed for processing reads, such as error correction and quality filters, as well as additional programs and pipelines that perform genome assemblies of reads generated by NGS platforms, producing complete genomes or scaffolds [3,4]. As a result of assembly reads, many contigs are produced. These reads or reference genomes can be used to order the contigs to produce a scaffold. Some regions in the scaffold have no assigned bases (A,C,T or G) due to the limitations of sequencing technology or assembly algorithms; these regions are called gaps and are usually represented by Ns [5–7].

Beyond commercial programs, such as CLC Genomic Workbench and Lasergene Suite, which have available options for finishing genome assemblies, including steps that fill gaps, open source programs are available. For example the open source programs G4ALL [8], GapCloser [3], GapFiller [6], and FGAP [9] use different approaches, such as paired reads or results of assemblies obtained with different software, to fill gap regions. The FGAP program was implemented in Matlab language and uses a draft of the assembly and a set of contigs that are mapped against genome draft to close gaps using BLAST algorithms. Both a fasta and a log file that report the filled gaps are generated at the end of the process. However, FGAP has no graphical interface [9].

G4ALL was implemented via JAVA programming language. The software has a graphical interface that allows the user to perform gap closure through manual curation of the scaffolds by comparing the BLAST results of the assembled contigs to the assembled scaffolds, similar to the GapBlaster method. G4ALL is useful for extending the contigs based on the overlap between them; however, it does not use contigs to close the gap regions [8].

GapCloser uses the information from paired reads to extend the sequences of contigs between gaps. Thus, the gaps can be closed or reduced [3]. Similar to GapCloser, the GapFiller program uses paired reads and is able to use data from different sequencing rounds simultaneously [6]. It is one of the available tools for closing gaps in prokaryotic and eukaryotic genomes of sizes up to ~100 Mb [10].

Genomes that have gaps may impair further studies because they may only partially represent an organism’s gene repertoire. Incomplete genomes can affect downstream analyses of genomic plasticity and comparative genomics [11].

Therefore, it is important to use complete genomes for comparative studies to properly characterize genome structure variations and gene content. This characterization allows the identification of genes that are 1) shared among all isolates and are thus useful for applied issues, such as vaccine and drug design [12]; 2) shared by some organisms, but not all studied organisms, and are thus useful for studying the reference lab activities for pathogenic bacteria [13,14]; and 3) present in a single isolate providing information regarding bacteria lifestyle [15].

Thus, this study presents a computational tool with a graphical user interface that helps reduce gaps through manual curation to increase the completion of genome assembly, rather than relying on the complete automation of this task.

## Materials and Methods

### Implementation

The GapBlaster was developed via JAVA programming language (http://java.sun.com/) using the paradigm of object orientation and the Swing library to create the visual resources (http://java.sun.com/docs/books/tutorial/uiswing).

Through the main interface of GapBlaster (S1 Fig), the user can input the scaffold and the contig files in FASTA format. After processing, another screen (S2 Fig) shows the alignment results. The user is then able to perform manual curation and select alignments that fill gaps confidently, as when the user finds a contig aligned in the gap flanks, closing the gap completely, as shown in S3 Fig.

GapBlaster performs five steps to identify possible gaps to be filled. All of the contigs obtained in the assembly are aligned against the draft genome or scaffold using BLAST Legacy [16], Blast+ or Mummer [17] based on user choice, and the alignment result is converted to the GapBlaster format. The contigs are subsequently ordered according to the mapping position in the scaffold. The program searches the alignments of the same contig that flank gap regions. A new ordination of the alignments is performed to determine the best option for gap closure. All identified alignments that fill gaps are presented to the user for evaluation (accepted or rejected) through the GapBlaster interface, and a log of changes made is generated. The selection of the alignment and the parameters can be defined by the user through the GapBlaster interface.

### Test data

To evaluate the GapBlaster program, analyses were conducted using two datasets: the first used sequencing data of *Corynebacterium pseudotuberculosis*, and the second was obtained from the GAGE (Genome Assembly Gold-Standard Evaluation) assembly of genomes [18].

*C*. *pseudotuberculosis* is a facultative intracellular gram-positive bacterium that causes caseous lymphadenitis (CLA), an infectious disease that affects small ruminants and belongs to CMNR group (Corynebacterium, Mycobacterium, Nocardia, and Rhodococcus) [19].

The sequencing of *C*. *pseudotuberculosis* was performed by an Ion Torrent PGM platform (Table 1). The reads (available in SRA database: SRR3312980) were assembled by a *de novo* strategy using SPADES version 3.1.0, with default parameters for Ion Torrent PGM data [20]. The scaffolds and contigs files produced in the assembly (available in https://sourceforge.net/projects/gapblaster2015/files/test_dataset/) were used as inputs in GapBlaster.

**Table 1:** Sequencing information of the genomes used in the analysis.

Organism	Platform	Library	Read Length	Insert Size	Number of Reads
*Corynebacterium pseudotuberculosis* 262	Ion Torrent PGM	Fragment	~220 bp	N/A	1765213
*Staphylococcus aureus* A-S391_USA300	Illumina	Paired-end	~101 bp	180 bp	1294104
*Staphylococcus aureus* A-S391_USA300	Illumina	Mate-Pair	~37 bp	3500 bp	3494070
*Rhodobacter sphaeroides 2*.*4*.*1*	Illumina	Paired-end	~101 bp	180 bp	2050868
*Rhodobacter sphaeroides 2*.*4*.*1*	Illumina	Mate-Pair	~101 bp	3500 bp	2050868

The GAGE dataset had the assemblies of the *Staphylococcus aureus* and *Rhodobacter sphaeroides* genomes, containing contigs and scaffolds generated by the following assemblers: Abyss, ABySS2, AllPaths-LG, Bambus2, MSR-CA, SGA, SOAPdenovo and Velvet for both organisms, whereas the CABOG was used for only *Rhodobacter sphaeroides* [18]. The data are available at http://gage.cbcb.umd.edu/, and the genome sequencing information can be seen in Table 1.

### GapBlaster

All contigs and scaffolds of the datasets were manually evaluated with GapBlaster version 1.1.1 to close gaps. In our analysis, we used one scaffold and one contig file for each organism/assembly, with the parameter Flank Length = 11 and the aligner Blast+ (the parameters in the GapBlaster should be set to reproduce our results). To close gaps, regions flanking the gaps (represented by Ns) were considered only when they had high identity (the threshold should be defined by the user).

### Gap closure comparison

To compare gap filling performance, GapBlaster, GapFiller and FGAP software were used in a gap closure analysis of the GAGE dataset and *C*. *pseudotuberculosis*.

The GAGE dataset with the mate-pair reads was analyzed with GapFiller [6] and FGAP [9] based on gap closure performance. Both types of software were used under default parameters, and the results were subsequently compared to GapBlaster.

The *C*. *pseudotuberculosis* genome was analyzed with FGAP only as GapFiller software requires paired-end libraries, and *C*. *pseudotuberculosis* was sequenced using fragmented libraries. The results of FGAP were compared to GapBlaster.

Additionally, GapBlaster was used to reduce gaps in the output files of FGAP and GapFiller software.

### Results evaluation

To validate the gap filling analysis, an in-house script was developed to evaluate the amount of gaps and Ns for each of the tests. The FASTA file (original scaffolds and the results of GapBlaster, FGAP, and GapFiller) was used as an input to count the number of gaps and their respective sizes. This script and a brief manual are available at https://sourceforge.net/projects/gapblaster2015/upload/scripts/.

To confirm if the gaps were correctly closed, the validation script of GAGE was used (http://gage.cbcb.umd.edu/results/gage-paper-validation.tar.gz). The input of this script was the reference genome (Table 2) and the original scaffold or gap-filled scaffold file.

**Table 2:** Information of the reference genomes used to validate the filled-in gaps.

Organism	*Corynebacterium Pseudotuberculosis 262*	*Staphylococcus Aureus* A-S391_USA300	*Rhodobacter Sphaeroides 2*.*4*.*1*
**Genome Size**	2325749	2872916	4628173
**GC Content**	52,17	0,3276	68,77
**Number of Chrs**	1	1	2
**Number of Plasmids**	0	0	5
**Genbank**	CP012022.1	CP007690.1	GCA_000273405.1
**150 pb Repeats**	8007	10709	38073
**250 pb Repeats**	6612	9460	35353

## Results and Discussion

The assembly results (number of bases and scaffolds) of the *C*. *pseudotuberculosis* genome produced by SPADES and the information concerning several assemblies of *S*. *aureus* and *R*. *sphaeroides* produced by various types of assemblers are shown in Table 3.

**Table 3:** Genome assembly information for *C*. *pseudotuberculosis* 262, *S*. *aureus* and *R*. *sphaeroides*.

Organism	Assembler	Bases (with N)	#Scaffolds
***C*. *pseudotuberculosis 262***	-------	**-------**	**-------**
	SPADES	2893857	4611
***S*. *aureus***	-------	**-------**	**-------**
	ABySS	3893185	5012
	ABySS2	3821622	125
	Allpaths-LG	2880676	19
	Bambus2	2862930	17
	MSR-CA	2872905	17
	SGA	3128388	546
	SOAPdenovo	2924135	175
	Velvet	2877995	173
***R*. *sphaeroides***	-------	**-------**	**-------**
	ABySS	5160167	2714
	ABySS2	5331930	480
	Allpaths-LG	4609785	38
	Bambus2	4428612	92
	CABOG	4259679	130
	MSR-CA	4498559	44
	SGA	5614693	2096
	SOAPdenovo	4627058	312
	Velvet	4615068	382

The results of the gap closure process for the *Corynebacterium* data assembled by SPADES are shown in Table 4.

**Table 4:** Gap closure results for the *Corynebacterium* genome.

	#Gaps	#N	#Gaps GB	#N GB	#Gaps FGAP	#N FGAP
***Corynebacterium pseudotuberculosis***	24	1794	11	931	5	360

Results of gap closure analysis of *Corynebacterium*, showing the #Gaps (amount of gaps) and #N (gap length); #Gaps GB and #N GB show the amount of remaining gaps and Ns, respectively, after the use of GapBlaster. The #Gaps FGAP and #N FGAP show the amount of remaining gaps and Ns, respectively, after the use of FGAP.

For *C*. *pseudotuberculosis* the amount of gaps was reduced from 24 to 11 with GapBlaster, and from 24 to 5, with FGAP. Gap length was also reduced for the *Corynebacterium* genome, as shown in Table 4. The *C*. *pseudotuberculosis* genome was sequenced using fragment libraries; thus, they could not be submitted to GapFiller.

The GAGE data of *S*. *aureus* and *R*. *sphaeroides* were assembled by several assemblers, and the results (contigs and scaffolds) were submitted to GapBlaster, FGAP and GapFiller. All assemblies of *S*. *aureus* revealed reductions in gaps and Ns when analyzed by GapBlaster. For *R*. *sphaeroides*, only the data for SGA did not show a reduction in gaps by GapBlaster (Table 5). It is important to highlight that GapBlaster allows manual curation; it allows less stringent criteria with careful manual evaluation, which is able to produce better results.

**Table 5:** Gap closure results for GAGE Assemblies.

***Staphylococcus aureus***	**#Gaps**	**#N**	**#Gaps GB**	**#N GB**	**#Gaps FGAP**	**#N FGAP**	**#Gaps GF**	**#N GF**
AbySS	66	55882	55	47614	45	51127	69	56355
AbySS2	33	9391	27	7780	17	4850	35	10003
Allpaths-LG	23	9875	20	9446	15	8755	40	10472
Bambus2	95	29201	93	29159	80	27459	98	30771
MSR-CA	81	10353	72	7868	47	7861	80	11651
SGA	654	300607	642	292067	634	298252	654	312284
SOAPdenovo	9	4857	8	4837	7	4708	9	5010
Velvet	128	17688	124	17473	94	15406	127	19863
***Rhodobacter sphaeroides***	***#Gaps***	***#N***	***#Gaps GB***	***#N GB***	***#Gaps FGAP***	***#N FGAP***	***#Gaps GF***	***#N GF***
AbySS	261	114525	261	114525	256	113886	306	118298
AbySS2	235	62570	233	62128	228	60323	290	68052
Allpaths-LG	90	21329	87	20733	82	19500	164	24001
Bambus2	85	57041	83	56402	80	55990	84	56930
CABOG	193	21547	192	20892	190	21065	191	25011
MSR-CA	356	32628	349	26189	347	31174	336	37494
SGA	938	1145600	938	1145600	930	1144955	930	1159235
SOAPdenovo	38	10461	37	9601	37	10097	38	11176
Velvet	427	86815	424	86785	404	86063	415	94150

Results of the gap closure process for the data produced by GAGE with several assemblers for *S*. *aureus* and *R*. *sphaeroides*. Showing the #Gaps (amount of gaps) and #N (gap length); #Gaps GB and #N GB show the amount of remaining gaps and Ns, respectively, after the use of GapBlaster. The #Gaps FGAP and #N FGAP show the amount of remaining gaps and Ns, respectively, after the use of FGAP. The #Gaps GF and #N GF show the amount of remaining gaps and Ns, respectively, after the use of GapFiller.

The FGAP and GapFiller programs were used to perform the gap closure step, and these results were compared with those obtained by GapBlaster (Table 5). GapFiller increased the numbers of gaps in most of the analyzed assemblies due the insert length, which was used to align against the reference sequences. In other cases, any gap that was closed had its length (the amount of Ns) increased, which occurred for the assemblies from SGA and SOAPdenovo for *S*. *aureus* and for the assemblies from SOAPdenovo for *R*. *sphaeroides*. Other results showed that GapFiller reduced the amount of gaps but increased their length (amount of Ns), which was observed for MSR-CA for *S*. *aureus* and CABOG, MSR-CA, SGA and for Velvet for *R*. *sphaeroides* (Table 5). Despite GapFiller having closed more gaps than GapBlaster for CABOG, MSR-CA, SGA and Velvet for *R*. *sphaeroides*, GapBlaster was superior to GapFiller. GapBlaster was able to fill more gaps and reduce the number of Ns in the sequences for nearly all GAGE assemblies, although it did not use paired reads.

FGAP filled more gaps than GapBlaster for all assemblies of the GAGE dataset. Nevertheless, GapBlaster filled more Ns than FGAP for ABySS and SGA for *S*. *aureus* and CABOG, MSR-CA and Velvet for *R*. *sphaeroides* (Table 5).

Despite FGAP performing the gap filling analysis automatically while GapBlaster performed the analysis manually, they achieved very similar results with respect to the number of gaps and N reductions for SOAPdenovo for *S*. *aureus* and Bambus2, CABOG, MSR-CA and SOAPdenovo for *R*. *sphaeroides* (Table 5).

FGAP showed better results for both the *C*. *pseudotuberculosis* and the GAGE datasets. We performed the gap filling analysis of the FGAP results with the original contigs of each organism and assembly through GapBlaster to determine whether GapBlaster could improve the results produced by FGAP. The results are shown in Table 6. Compared with the FGAP results, GapBlaster improved 55.55% of all assemblies of the GAGE dataset and *C*. *pseudotuberculosis*. GapFiller was not used for this comparison of *Corynebacterium* data because only a fragment library was available for this organism.

**Table 6:** Comparison of the original results of FGAP and after manual curation with GapBlaster.

***Staphylococcus aureus***	**#Gaps FGAP**	**#N FGAP**	**#Gaps after GB**	**#N after GB**
AbySS	45	51127	41	45439
MSR-CA	47	7861	46	6359
SGA	634	298252	629	290825
***Rhodobacter sphaeroides***	**#Gaps FGAP**	**#N FGAP**	**#Gaps after GB**	**#N after GB**
AbySS2	228	60323	227	60040
Allpaths-LG	82	19500	81	19494
Bambus2	80	55990	79	55402
CABOG	190	21065	188	19568
MSR-CA	347	31174	343	25592
SOAPdenovo	37	10097	36	9237
	**#Gaps FGAP**	**#N FGAP**	**#Gaps after GB**	**#N after GB**
***Corynebacterium pseudotuberculosis***	5	360	3	251

The results produced by FGAP were used as input for GapBlaster, and the organism/assemblies that were improved are shown. The #Gaps FGAP and #N FGAP show the amount of gaps and Ns, respectively, for the results of FGAP. The #Gaps **after** GB and #N **after** GB show the amounts of remaining gaps and Ns, respectively, after the use of GapBlaster.

GapBlaster improved the results of FGAP for *C*. *pseudotuberculosis* in that it reduced the number of gaps from 5 to 3. Therefore, GapBlaster improved the gap filling results for several assemblies for *S*. *aureus* and *R*. *sphaeroides*, as shown in Table 6. This analysis shows that despite its usefulness for closing gaps through its GUI, GapBlaster is also useful for gap filling when used in combination with another tools.

Similar to the analysis of the FGAP results, we conducted an evaluation of the GapFiller output files and the original contigs of each organism/assembler of the GAGE dataset via GapBlaster. Compared with the GapFiller results, GapBlaster improved 70.58% of all assemblies of the GAGE dataset (Table 7).

**Table 7:** Comparison of the original results of GapFiller and after manual curation with GapBlaster.

***Staphylococcus aureus***	**#Gaps GF**	**#N GF**	**#Gaps after GB**	**#N after GB**
AbySS	69	56355	66	54837
AbySS2	35	10003	30	8741
Allpaths-LG	40	10472	39	10455
Bambus2	98	30771	97	30725
MSR-CA	80	11651	76	9794
SGA	654	312284	646	307095
***Rhodobacter sphaeroides***	**#Gaps GF**	**#N GF**	**#Gaps after GB**	**#N after GB**
AbySS	306	118298	304	118287
AbySS2	290	68052	288	67740
Allpaths-LG	164	24001	163	23780
CABOG	191	25011	190	24336
MSR-CA	336	37494	333	33590
SGA	930	1159235	929	1159162

The results produced by GapFiller were used as input for GapBlaster, and the organism/assemblies that were improved are shown. The #Gaps GF and #N GF show the amount of gaps and Ns, respectively, in the results of GapFiller. The #Gaps **after** GB and #N **after** GB show the amount of remaining gaps and Ns, respectively, after the use of GapBlaster.

GapBlaster improved the results of GapFiller for almost all of the CAGE data (Table 7). The best gap filling results were ABySS2 and SGA for *S*. *aureus*, where the gaps decreased from 35 to 30 and 654 to 646, respectively (Table 7). Beyond being a very useful tool with an interface for manual curation, GapBlaster is a valuable open source program that can be used with other tools in the gap filling analysis to produce more complete genome drafts.

To evaluate the accuracy of the closed gaps, all results produced by GapBlaster, FGAP, GapFiller and the original files (scaffolds) were aligned against their respective genome reference (Table 2). The results show that all of the files produced in the gap filling analysis showed similar alignment percentages with the original files, which confirms that the bases introduced in the filled gaps were correct (S1 Table).

Despite the three methods (Blast+, Blast Legacy, and Mummer) implemented in GapBlaster, we used only Blast+ to fill gaps as this method is the same used for FGAP software. However, we tested all of the algorithms for GAGE data, and Blast Legacy and Blast+ presented similar results (S2 Table). The comparisons of the features of GapBlaster, FGAP and GapFiller helped to identify the main advantage of GapBlaster, the graphical interface, which uses contigs to fill gaps and allows manual curation (Table 8).

**Table 8:** Comparison of the features of GapBlaster, FGAP and GapFiller.

Features	GapBlaster	FGAP	GapFiller
Alignment method	Blast+ or Blast Legacy or Mummer	Blast+	Bowtie or BWA
Set Flank Alignment	Yes	Yes	Yes
Allow Manual Curation	Yes	No	No
Perform Automatic Analysis	Yes	Yes	Yes
Based on paired-reads	No	No	Yes
Use contigs to fill gaps	Yes	Yes	No
Graphical interface	Yes	No	No
Improve gap filling results of other softwares	Yes	Not tested	Not tested
Correctly fill gaps?	Yes	Yes	Yes

## Conclusions

Despite the efficiency of tools such as FGAP and GapFiller, the gap closure process continues to be a step that requires manual curation for the acquisition of high quality results, such as those presented by GapBlaster, the use of which is simplified by the graphical interface.

GapBlaster revealed improved gap filling performance using contigs compared to GapFiller for nearly all data evaluated despite the use of paired reads in GapFiller.

In addition to presenting better results, the GapBlaster program has the advantage of introducing fewer errors, based on the ability of the interface to allow the user to decide if a gap is filled properly. As an alternative, GapBlaster can be used in addition to other gap closer programs to facilitate genome completion through manual manipulation, as was shown in the analysis of the GapBlaster program to improve the results of FGAP and GapFiller.

## Supporting Information

S1 Fig GapBlaster main interface. The main graphical interface through which the user can input the contigs and scaffold files and set the alignment preferences.
(TIF)

S2 Fig Alignment interface. The screen shows the results of the alignment of a contig against a scaffold. All alignments produced are listed. The user can check if the alignments are correct and select them.
(TIF)

S3 Fig Selected Alignment. The aligned contig filled the gap with high accuracy due to the high identity found in the gap flanks.
(TIF)

S1 Table GapBlaster, FGAP and GapFiller accuracy. This table shows information about the percentage of bases aligned against the GapBlaster, FGAP, GapFiller results and the original scaffolds to the reference genome to evaluate if the filled gaps introduced the correct bases in the analysis.
(XLS)

S2 Table GapBlaster algorithm comparison. Comparison of the three alignment algorithms implemented in GapBlaster (Blast+, Blast Legacy, Mummer) to evaluate the number of alignments identified, closed gaps and N removed after the gap filling process.
(XLS)

## References

[1] Liu L, Li Y, Li S, Hu N, He Y, Pong R, et al Comparison of next-generation sequencing systems. J Biomed Biotechnol. 2012;2012: 251364 10.1155/2012/251364 22829749 PMC3398667

[2] Wojcieszek M, Pawełkowicz M, Nowak R, Przybecki Z. Genomes correction and assembling: present methods and tools. SPIE Proc. 2014;9290: 92901X 10.1117/12.2075624

[3] Luo R, Liu B, Xie Y, Li Z, Huang W, Yuan J, et al. SOAPdenovo2: an empirically improved memory-efficient short-read de novo assembler. 2012; 1–6. 10.1186/2047-217X-1-18 PMC3626529 23587118

[4] Zerbino DR, Birney E. Velvet: algorithms for de novo short read assembly using de Bruijn graphs. Genome Res. 2008;18: 821–9. 10.1101/gr.074492.107 18349386 PMC2336801

[5] Boetzer M, Henkel C V, Jansen HJ, Butler D, Pirovano W. Scaffolding pre-assembled contigs using SSPACE. Bioinformatics. 2011;27: 578–9. 10.1093/bioinformatics/btq683 21149342

[6] Boetzer M, Pirovano W. Toward almost closed genomes with GapFiller. Genome Biol. BioMed Central Ltd; 2012;13: R56 10.1186/gb-2012-13-6-r56 22731987 PMC3446322

[7] Ekblom R, Wolf JBW. A field guide to whole-genome sequencing, assembly and annotation. Evol Appl. 2014;7: 1026–1042. 10.1111/eva.12178 25553065 PMC4231593

[8] Ramos RTJ, Carneiro AR, Caracciolo PH, Azevedo V, Schneider MPC, Barh D, et al Graphical contig analyzer for all sequencing platforms (G4ALL): a new stand-alone tool for finishing and draft generation of bacterial genomes. Bioinformation. 2013;9: 599–604. 10.6026/97320630009599 23888102 PMC3717189

[9] Piro V, Faoro H, Weiss V. FGAP: an automated gap closing tool. BMC Res Notes. 2014; 1–5. 10.1186/1756-0500-7-371 24938749 PMC4091766

[10] Paulino D, Warren RL, Vandervalk BP, Raymond A, Jackman SD, Birol I. Sealer: a scalable gap-closing application for finishing draft genomes. BMC Bioinformatics. BMC Bioinformatics; 2015;16: 230 10.1186/s12859-015-0663-4 26209068 PMC4515008

[11] Touchman J. Comparative Genomics. In: Comparative Genomics. Nature Education Knowledge 3(10):13 [Internet]. 2010 Available: http://www.nature.com/scitable/knowledge/library/comparative-genomics-13239404

[12] Seib KL, Zhao X, Rappuoli R. Developing vaccines in the era of genomics: a decade of reverse vaccinology. Clin Microbiol Infect. 2012;18: 109–116. 10.1111/j.1469-0691.2012.03939.x 22882709

[13] Theodore MJ, Anderson RD, Wang X, Katz LS, Vuong JT, Bell ME, et al Evaluation of new biomarker genes for differentiating Haemophilus influenzae from Haemophilus haemolyticus. J Clin Microbiol. 2012;50: 1422–1424. 10.1128/JCM.06702-11 22301020 PMC3318567

[14] Tatti KM, Loparev VN, Ranganathanganakammal S, Changayil S, Frace M, Weil MR, et al Draft genome sequences of Bordetella holmesii strains from blood (F627) and nasopharynx (H558). Genome Announc. 2013;1: e0005613 10.1128/genomeA.00056-13 23516195 PMC3622985

[15] Mira A, Martín-Cuadrado A. The bacterial pan-genome: a new paradigm in microbiology. Int Microbiol. 2010; 45–57. 10.2436/20.1501.01.110 20890839

[16] Altschul SF, Gish W, Miller W, Myers EW, Lipman DJ. Basic local alignment search tool. J Mol Biol. 1990;215: 403–410. 10.1016/S0022-2836(05)80360-2 2231712

[17] Delcher A, Kasif S. Alignment of whole genomes. Nucleic Acids Res. 1999;27: 2369–2376. Available: http://nar.oxfordjournals.org/content/27/11/2369.short 10325427 10.1093/nar/27.11.2369 PMC148804

[18] Salzberg SL, Phillippy AM, Zimin A, Puiu D, Magoc T, Koren S, et al GAGE: A critical evaluation of genome assemblies and assembly algorithms. Genome Res. 2012;22: 557–67. 10.1101/gr.131383.111 22147368 PMC3290791

[19] Dorella FA, Pacheco LG, Oliveira SC, Miyoshi A, Azevedo V. Corynebacterium pseudotuberculosis: microbiology, biochemical properties, pathogenesis and molecular studies of virulence. Vet Res. 2006;37: 201–218. 10.1051/vetres 16472520

[20] Bankevich A, Nurk S, Antipov D, Gurevich A a., Dvorkin M, Kulikov AS, et al SPAdes: A New Genome Assembly Algorithm and Its Applications to Single-Cell Sequencing. J Comput Biol. 2012;19: 455–477. 10.1089/cmb.2012.0021 22506599 PMC3342519

