# Supplementary figures and images for: GapBlaster—A Graphical Gap Filler for Prokaryote Genomes
Source: PLoS One. 2016 May 12;11(5):e0155327. doi: 10.1371/journal.pone.0155327 (PMC4865197; doi:10.1371/journal.pone.0155327)

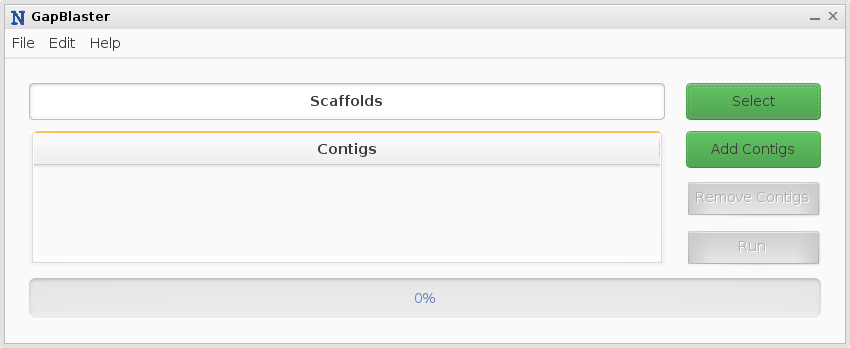

Supplement: S1 Fig — The main graphical interface through which the user can input the contigs and scaffold files and set the alignment preferences. (TIF) [file pone.0155327.s001.tif]

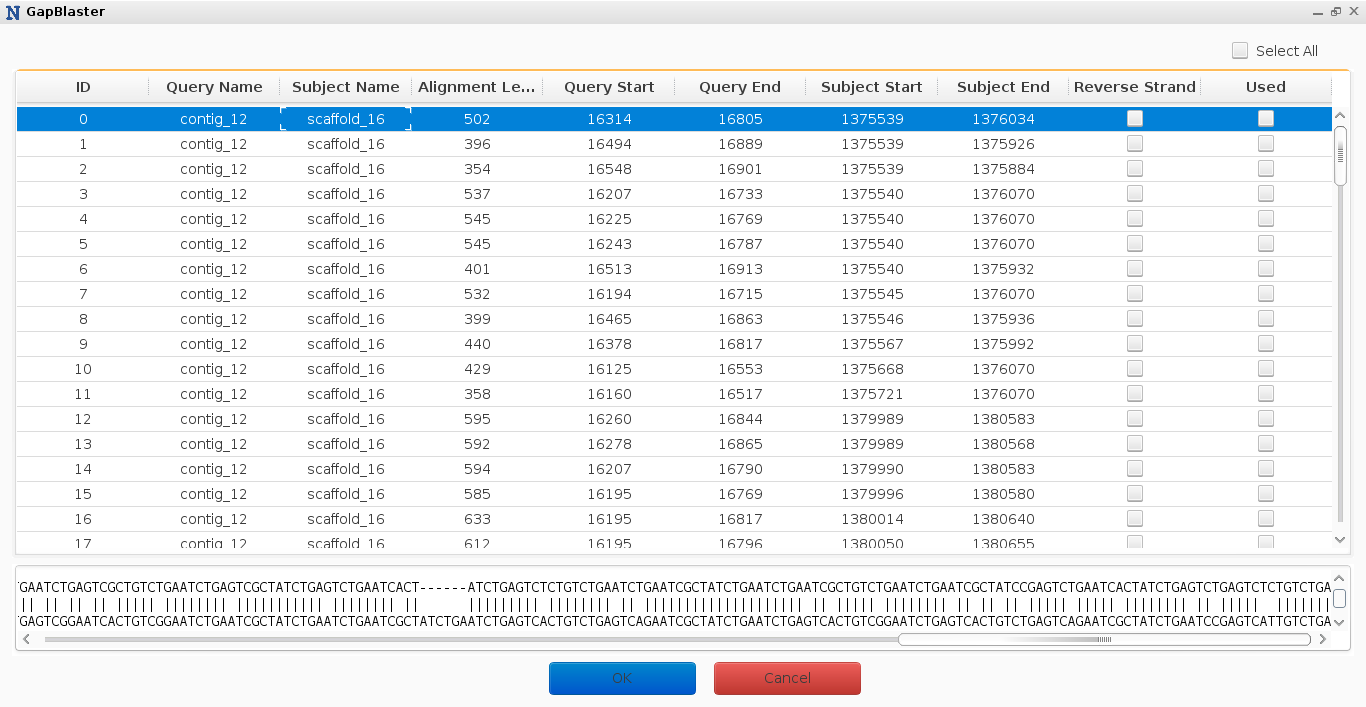

Supplement: S2 Fig — The screen shows the results of the alignment of a contig against a scaffold. All alignments produced are listed. The user can check if the alignments are correct and select them. (TIF) [file pone.0155327.s002.tif]

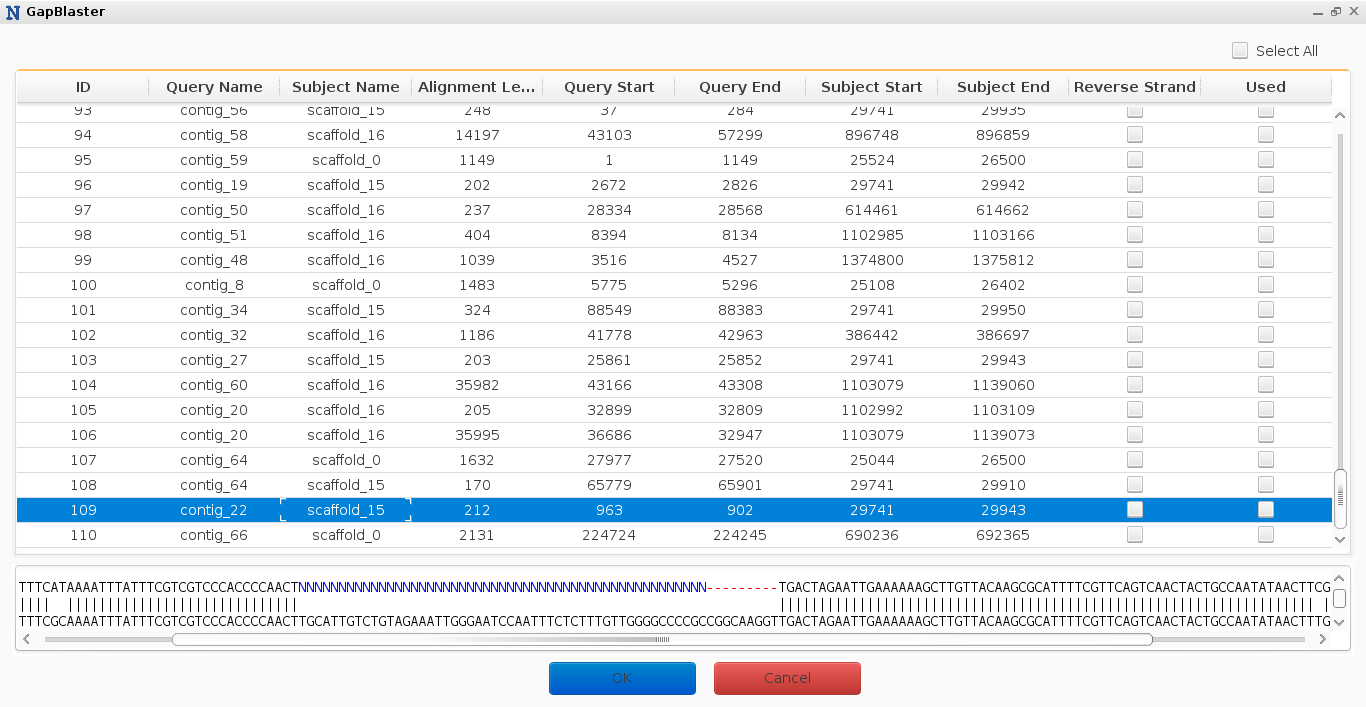

Supplement: S3 Fig — The aligned contig filled the gap with high accuracy due to the high identity found in the gap flanks. (TIF) [file pone.0155327.s003.tif]
